# Account of an Epidemic Bilious Pleurisy, Which Occurred in Appling, Columbia Co., Georgia, in the Fall and Winter of 1830

**Published:** 1831

**Authors:** Allen Kimball


Art. II.—*Account of an Epidemic Bilious Pleurisy which occurred in Appling, Columbia County, Georgia, in the fall and winter of* 1830, by Dr. Allen Kimball.

*Appling, January* 18, 1830.

Dear Sir,—I take up my pen to give you a few cursory remarks on an epidemic bilious pleurisy which appeared in this vicinity since the month of November. The late fall has been noted for its long and continued drouth; together with considerable heat. From August until near the close of November, are scarcely ever known to have occurred fewer showers or less rain. Towards the close of the present month the weather changed from dry and warm to cold and showery; to which, in a few days, succeeded heavy rains, cold winds, frosts, and ice. The thermometer frequently ranged from 20 to 10 degrees, a degree of cold very unusual for this climate at this season. This sudden and unexpected change was soon followed by serious pulmonary and catarrhal affections. The first cases which occurred had their origin under circumstances somewhat peculiar, and will remind you in some particulars, of the origin of fatal fever of the old Baileys, and is highly corroborative of the explanation given of the true cause of that disease by Bancroft.

These cases were consequent on a nuptial and convivial association of a number of friends. The night was unusually cold, the room, (I am creditably informed,) was close and highly heated. After the usual routine of ceremony, the company indulged freely in dancing and other amusements, until they were excited to a high degree of heat and perspiration. In this situation they frequently retired from the room to cool and refresh themselves in the open air; thus exposing, to the injurious effects of cold, a system relaxed and debilitated by an atmosphere, com-composed (beside the impurities consequent on a crowded and ill-ventilated apartment) of an endless variety of vinous, spiritous, and animal fumes, all tending to impede respiration, to prevent the arterialization of the blood, and to produce morbid and preternatural excitements of the glandular and cutaneous systems. These cases partook of the general character of the epidemic that succeeded. Those that were under my care were attacked within a few days, of a "*catarrhus* a *frigore*," a dull and heavy pain over the brow and within the frontal sinus; frequent paroxysms of a deep, dry, and hollow cough; expectoration slight, occasionally stained with blood, or a light thin straw colored matter; eyes yellow; tongue rather red except its margin which was of a whiter aspect. In some a slight pain in the right side, particularly when coughing. These symptoms continued to increase from two to five days, when the cough became almost insupportable, no expectoration; tongue yellow, and bilious; pain, distressing in the frontal region and along the cervical column, the right side and sternal region. In the latter, most complained of a full and uneasy sensation, and others of a difficulty in breathing. Pulse in some, frequent and moderately full; in most, however, soft and yielding readily to pressure; a few far below the healthy standard. In all there was a tendency to mental alienation, varying from the mildest form of intellectual derangement to *insanity*. Some were wild and restless, tossing from side to side of the bed, imagining upon every secret conversation that their friends were anticipating inevitable death; while others were typhoid and did not complain at all, as complete somnolency seemed to prevent the exercise of the intellectual faculties. They would frequently attempt to reply readily to enquiries; but, would as soon seem to forget, and hesitate, and the answer would be entirely irrelative to the question. At this stage there were uniformly flushes and livid spots of the right cheek, and partial *subsultus tendinum*. There evidently seemed to have been two forms and termination of this fever. The difference consisted in this; the first there was a wildness, restlesness, &c., pain in the side, and pulse not very full but beat from 80 to 100, while in the latter there was not so much pain in the side, complete somnolency and stupor; pulse from 45 to 60, and perhaps, might be termed a very quick pulse. One of these cases was bled, to eight ounces, by my directions, on the first approach of the stupor, but the unfavorable symptoms were greatly increased. On account of the bilious symptoms, he took an active mercurial cathartic every day, combined with anodynes, and sudorificks, but the stupor and prostration only seemed to be the more speedily superadded. About the fifth day from the first appearance of these symptoms, on consultation, I reluctantly consented to a second bloodletting, on the suggestion that it was a *fac simile* of Rush's "frebris synocha," with suffocated excitement. I bled him myself, from six to eight ounces; the blood partook very slightly of the inflammatory buff. All the above symptoms were exasperated—a twitching of the muscles of the left side supervened on the lapse of a few hours, which was soon followed by a complete paralysis of that side, accompanied by the most violent convulsions, which returned at intervals of three to four hours. I immediately directed the whole scalp enveloped with a blister, administered sulph., quinæ, and carb. of amonia, and applied external frictions of mustard and capsic annuum. The blister drew and discharged immensely; in proportion to that evacuation the faculties of the mind returned, and a speedy cure was effected.*

---

* I followed here a practice, to arrest and mitigate the violence of the convulsion, which is objected to by good authority, viz; at the approach of the fit, to have the patient's limbs held fast by strong assistants.

The few cases of this description which I afterwards saw were not bled, but treated by frictions, blisters, calomel, nitre, and quinine and they all recovered speedily. The purgative preparation was as follows:

Calomel, 12 to 16 grs.
Nit Potassa, 16 "
Sulph. quinine, 6 "
Dov. powders, 12 "
Pulv. iv.

One to be given every two hours, and passed off by sen. and cream tartar infusion.

If the bowels were difficult to operate on and no irritability of the stomach, the dov. powder was omitted, and if much gastric excitability was present, and the pectoral affections abated, senna and manna were substituted for the infusion. The purgative was followed by flaxseed tea, and other diluents, as much as the stomach would bear.

Those that exhibited a more inflammatory diathesis,* varied in some essential points of practice, but did not so extensively as might be imagined. In the treatment of more than twenty cases that ran a violent and unyielding course, seven were not bled, but treated by purgatives, blisters, sudorifics, and expectorants. All recovered speedily. Seven were bled once only and recovered by the foregoing rules. They were generally bled from eight to ten ounces. The remainder were bled twice, and none more than thrice; one died. Of two cases of pregnancy one was followed by abortion. I very especially remarked one peculiarity, that those cases which partook most of the bilious type, did not bear bleeding with impunity, yet they did purgation, and *vice versa;* and it is also here worthy of remark, that those, who were attacked within twenty or

* Dr. Thomas informs us that it is no unusual occurrence, in cold latitudes, to find typhus complicated with topical inflamations of the thoracic viscera, constituting pneumonia typhode. In such cases he assures us that he has seen venesection detrimental in several instances where it seemed to destroy the patients. One class of the epidemic which I mentioned in the first part of this letter did unquestionably partake deeply of typhus, and venesection most assuredly proved injurious. Dr. Rush says, that a few cases of the epidemic influenza, of 89, &c., terminated in "tedious and dangerous typhus."

twenty-five days from the commencement of the epidemic, assumed a bilious type, while those of a subsequent origin, were but slightly associated with that type. To the first class, mercurial cathartics were daily given, but to the latter class, I scarcely gave a single dose of calomel. It is to the former of these cases that my remarks are more particularly directed. As expectorants, the oxymel of squills, seneka, flaxseed, and particularly the asclepias decumbens, were highly efficacious, at the same time the violence of the cough was controlled by small anodynes. I have long since been convinced of the great influence of warm climates over the hepatic system, and am warranted in the assertion, by every observant practitioner of Georgia, that all cases of *pleurisy* which do occur on sudden changes from heat to cold, partake of the bilious type, while those that occur after this system has been for a time under the influence of a cold temperature assume the type of northern pleurisies.

Heat relaxes the liver, and increases its secretions; cold has a contrary influence, rendering its circulation more slow, and suppressing its secretions. If, after long exposure to heat, the system be suddenly exposed to atmospheric changes, sufficient to produce pulmonary disease, the liver will partake in the disease, because it is already in an excited state; and the peculiar symptoms of biliary derangement will shew themselves.

Bichat says "cold atmosphere confines the functions of the skin, and the internal secretions are more abundant." Daily experience disproves this. In warm seasons and climates, the biliary secretions are increased, and vice versa. We must have, therefore, a perverted function of this organ, as a consequence of sudden vicissitudes. Uniformity of climate, is one of the most efficacious remedial agents to the southern bilious invalids.

It is very difficult to give the pathology of this typhoid form of disease. Many attribute the stupor to cerebral oppression. If this were the case, blood-letting should be an efficacious remedy. Why may we not attribute it to the imperfect oxygenation of the blood, in consequence of the congestion of the capillary system of the lungs? That this congestion does take place, we infer from the deep, dry cough, or the expectoration of mucus, tinged with blood, perhaps blood itself. Although the heart does not refuse to circulate venous blood, yet, the appearance of this blood in the arterial system, never fails to produce paralysis and stupor. All authors who have written on the subject, concur in assigning to carbonic acid gas, a sedative influence. Dr. Leonard says, that dilute carbonic acid reduces the frequency of the pulse, with as much certainty as digitalis, producing nausea, vomiting, and loss of appetite. In this way, as a late graduate of Transylvania University has contended, the most prominent symptoms of the cold stage of masmatic fevers, viz: a "darker hue of the blood, and a weaker action of the heart, may be imitated by the action of carbonic acid upon the animal economy. It is easy to imagine that this condition of the lungs would prevent the arterialization of the blood, and that the brain, from its delicacy, would be one of the first to feel its influence.

This view is directly confirmed by the symptoms we often meet with in pneumonia typhodes, which, very frequently, closely resemble the symptoms of the disease in question. At the same time, it may be borne in mind, that the lungs may be but one of the emunetories of carbon from the system, and that, very probably, the liver and kidneys serve a similar purpose. When the secretion of urine is suspended, a train of symptoms supervenes, not unlike analogous, in many respects, to those of the present disease. May we not therefore, in this instance, be justified in supposing, that the symptoms of cerebral oppression arise from the imperfection of that process by which venous is changed into arterial blood?

